# Role of remote ischaemic conditioning in fracture healing and orthopaedic surgery—a systematic review and narrative synthesis

**DOI:** 10.1186/s13018-025-05772-6

**Published:** 2025-05-07

**Authors:** Alison Buck, Tao Wang, Sheharyar S. Baig, Arshad Majid, Ali N. Ali

**Affiliations:** 1 MRes, Sheffield Teaching Hospitals NHS Foundation Trust, University of Sheffield, Sheffield, UK; 2 Department of Neuroscience, Sheffield Institute for Translational Neurosciences, University of Sheffield, Sheffield, UK; 3 Department of Neuroscience, Geriatrics and Stroke, Sheffield Institute of Translational Neuroscience, Royal Hallamshire Hospital, University of Sheffield, Glossop Rd, Sheffield, S10 2 JF UK

**Keywords:** Remote ischaemic conditioning, Ischaemia, Stroke, Myocardial infarction, Elective surgery, Emergency surgery

## Abstract

**Introduction:**

Remote ischaemic conditioning (RIC) involves the use of controlled and transient ischemia and reperfusion cycles, commonly of the upper or lower limb, to mitigate cellular damage from ischaemic events. Studies have demonstrated that RIC may have anti-inflammatory and cardiovascular protective effects and thus could represent a novel therapeutic strategy to improve outcomes following orthopaedic surgery. This review aimed to comprehensively describe the current pre-clinical and clinical evidence for RIC in orthopaedics.

**Methods:**

MEDLINE and EMBASE via OVID (1966—March 2024) were searched using a systematic search strategy for randomised controlled trials (RCTs) investigating the effects of RIC on fracture, bone healing, and orthopaedics. Both pre-clinical and clinical RCTs were included.

**Results:**

Three pre-clinical RCTs (comprising of 198 rats in models of experimental fracture) met the inclusion criteria. These showed that RIC was associated with enhanced callus formation (volume and biomechanical strength) post-fracture, reduced oxidative stress and upregulated osteoblastic activity. Sixteen clinical RCTs, involving 628 patients, investigated RIC in 6 different elective orthopaedic procedures (knee, lower limb, cervical, shoulder, general, hip fracture). RIC protocols varied in cycle frequency, duration, and pressure, but all were given as a single dose at induction of anaesthesia. Significant results included reductions in oxidative stress, improved cerebral and peripheral oxygenation, and reduced pain scores and analgesia use. Only 1 study (n = 648) evaluated RIC in acute hip fracture and demonstrated an early cardioprotective effect.

**Conclusion:**

The potential therapeutic effects of RIC in orthopaedic surgery is supported by preliminary evidence from pre-clinical and clinical studies. Trials to date are largely small but warrant investigation in well-powered multicentre RCTs. There are still many unanswered questions about the optimal RIC parameters (cuff pressure, frequency and duration) in orthopaedic surgery and determining which patients may benefit most from this therapy.

**Supplementary Information:**

The online version contains supplementary material available at 10.1186/s13018-025-05772-6.

## Background

Orthopaedic surgery is a common treatment option for acute and chronic musculoskeletal disorders [1]. Rates of bone fractures are rising [2] particularly in the elderly, and along with arthritis and pain syndromes contribute to adult-onset disability [2]. Consequently a third of the population suffer pain, stiffness and restricted movement impacting negatively on quality of life [3]. In the UK’s National Health Service (NHS), 25% of all surgical interventions are for musculoskeletal conditions with orthopaedic procedures accounting for 16.1% of the total cost of surgery [4]. An ageing and multimorbid population increase the risk of post-surgical complications including myocardial infarction, stroke, delayed healing, and infection [5]. Hip fractures in particular require hospitalisation and surgical repair [6] and affect 70,000 individuals in the UK annually, costing an estimated £1.1 billion [7] and is expected to double by 2040 [8, 9]. Mortality after hip fracture remains high at 6.1% in the first month, rising to 33% at one year in the UK. Thus, there is an unmet need for interventions that mitigate the risk of such post-surgical complications [9].

**Fig. 1:**
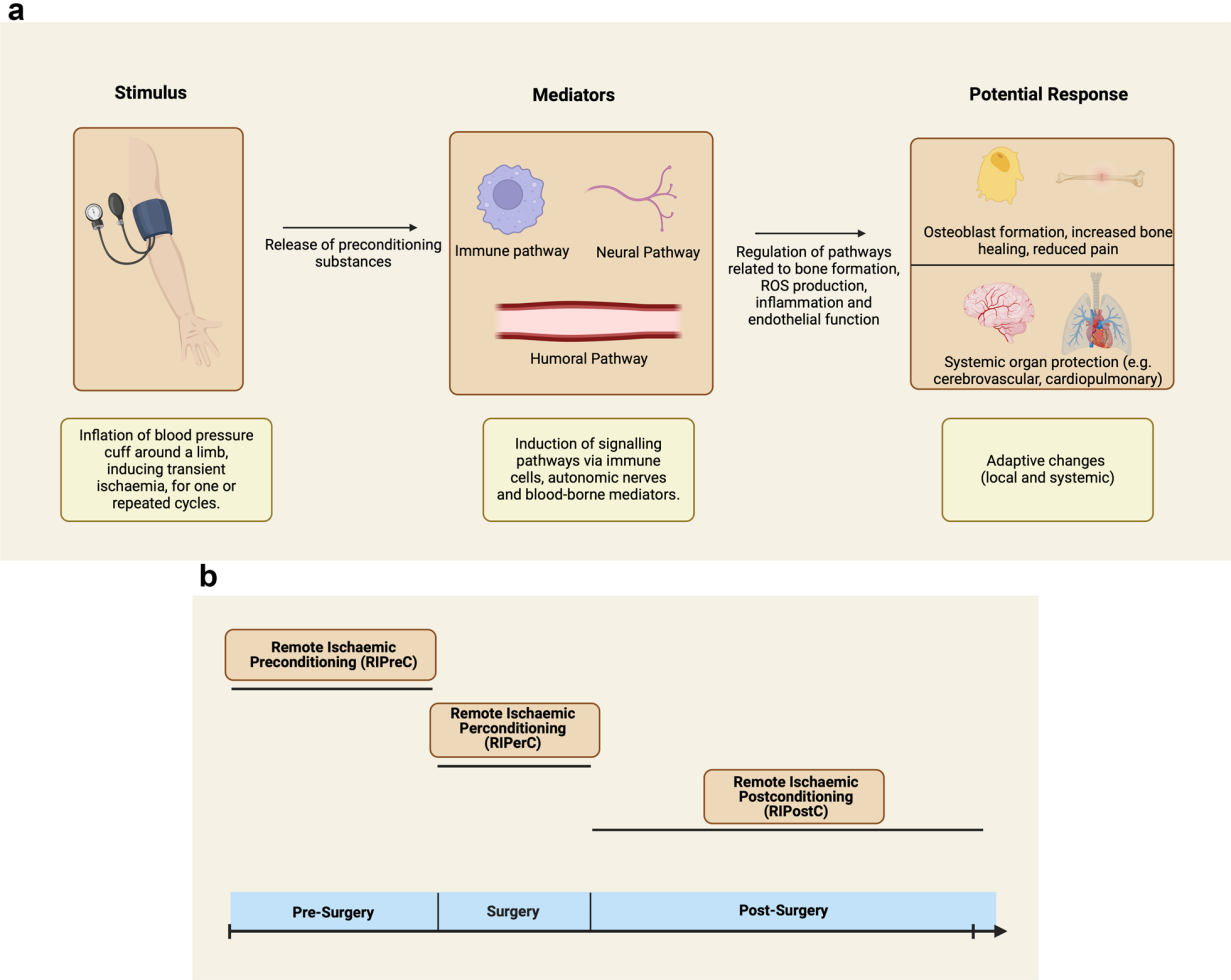
**a** Postulated mechanisms of action of remote ischaemic conditioning (RIC). **b** Different paradigms of remote ischaemic conditioning (RIC) delivery

Remote ischaemic conditioning (RIC) is a technique which induces intermittent ischaemia of the upper or lower limb, through inflating a pressure cuff above systolic blood pressure for intervals that avoid physical injury, but trigger a number of intrinsic protective mechanisms [10] (Fig. 1a). RIC was first shown to reduce infarct size in animal models of myocardial infarction in the 2000 s [11]. Since then, RIC has been studied in ischaemia–reperfusion injury of other organs such as the brain, kidney and liver, as well as for conditions such as sepsis and renal failure [12, 15]. Depending on timing of RIC relative to ischaemia, RIC is referred to as remote ischaemic preconditioning (RIPreC), perconditioning (RIPerC) or post-conditioning (RIPostC) [12] (Fig. 1b).

The underlying mechanism of action of RIC is thought to be mediated via humoral (release of blood-based signalling molecules such as nitric oxide), neuronal (activation of peripheral and central autonomic fibres) and immunoregulatory (suppression of pro-inflammatory cytokine production) pathways [16, 19]. The downstream effects of these, included improved mitochondrial and endothelial function may increase resilience to future bouts of ischaemia, both locally and systemically. [20, 21]. Furthermore, RIC has also been postulated to have effects on bone repair mechanisms and pain modulation in preclinical models and in clinical studies [22, 23] of fracture and musculoskeletal injury. As such, RIC may be a promising, low-cost adjuvant therapy in elective and emergency orthopaedic interventions.

This systematic review represents a comprehensive and contemporaneous review of the preclinical and clinical evidence to date that investigates its use in fracture healing and orthopaedic surgery.

## Methods

This systematic review followed the PRISMA reporting guidelines [24] (Additional file 1).

### Inclusion and exclusion criteria

Studies were included if they evaluated the effects of RIC on fracture healing, trauma, hip fracture or orthopaedic surgery. Only randomised controlled trials (RCTs) were included, both preclinical and clinical. We included all studies independent of the protocol of RIC used or their primary and secondary outcomes. Only articles written in English were included.

### Search strategy

The following electronic databases were searched from 1966 to March 2024: MEDLINE via OVID and EMBASE via OVID. Subject heading and free text terms relating to RIC (e.g. ischaemic conditioning, remote ischaemic conditioning, preconditioning, perconditioning, postconditioning), fracture (e.g. trauma, bone injury, fracture, hip fracture, break, fragility, bone healing), and orthopaedics (e.g. musculoskeletal, orthopaedics, trauma, ligament, meniscus, elective, emergency, operative, tissue, muscle, cartilage) were used to produce a search strategy for OVID MEDLINE (Additional file 2). This was adjusted using Boolean operators for EMBASE. Reference lists of included studies and reviews were scanned for relevant additional articles.

### Study selection and data extraction

The initial search results were reviewed independently by 2 authors (AB and AA), duplicate and irrelevant articles were removed from screening titles and abstracts. Full texts of the remaining articles were then reviewed for final inclusion, and data extracted into a predesigned spreadsheet. This included author details, study design, population or animal models, intervention details (timing of RIC, pressure protocols used, limbs conditioned), outcome measures reported, and clinical findings. Disagreements on study inclusion or outcomes were adjudicated by a third reviewer (TW).

### Study quality assessment

Two reviewers (AB and AA) independently reviewed each study. Preclinical studies were assessed using the SYRCLE’s tool for assessing risk of bias [25] composed of 10 items including: allocation sequence generation; similarity of baseline characteristics; allocation concealment; housing of animals; investigator blinding; random outcome assessment; blinding of assessments; completeness of data collection; selectivity or reporting and other sources of bias. Reporting of items in study manuscripts or protocols accrue a point each, with a maximum score of 10. Clinical studies were reviewed using the PEDro scale [26], a 10-item checklist addressing similar concepts based on the following: specification of eligibility criteria; randomisation; concealment of allocation; similarity of group baseline characteristics, subject blinding; blinding of therapists and assessors; completeness of data collection; proportion of allocated individuals receiving intended treatments or inclusion of ‘intention to treat’ analyses; between group statistical comparisons; and provision of measures of variability in outcome measures. Item 1 (eligibility criteria) of the PEDro is not scored, but presence of other quality markers accrue a point each so that a total of 10 is achieved for the highest quality studies. Scores of 0–3 are considered ‘poor’; 4–5 ‘fair’, 6–8 ‘good’ and 9–10 ‘excellent’.

### Data analysis and narrative review

Study characteristics and outcomes were qualitatively synthesised and summarised in tabular form. Due to the heterogeneity of study populations, RIC protocols and outcomes assessed we were unable to perform meta-analyses of outcome measures. We thus undertook a narrative synthesis of the available evidence using the framework published by the Cochrane Consumers and Communication Review Group [27].

## Results

### Study selection

**Fig. 2:**
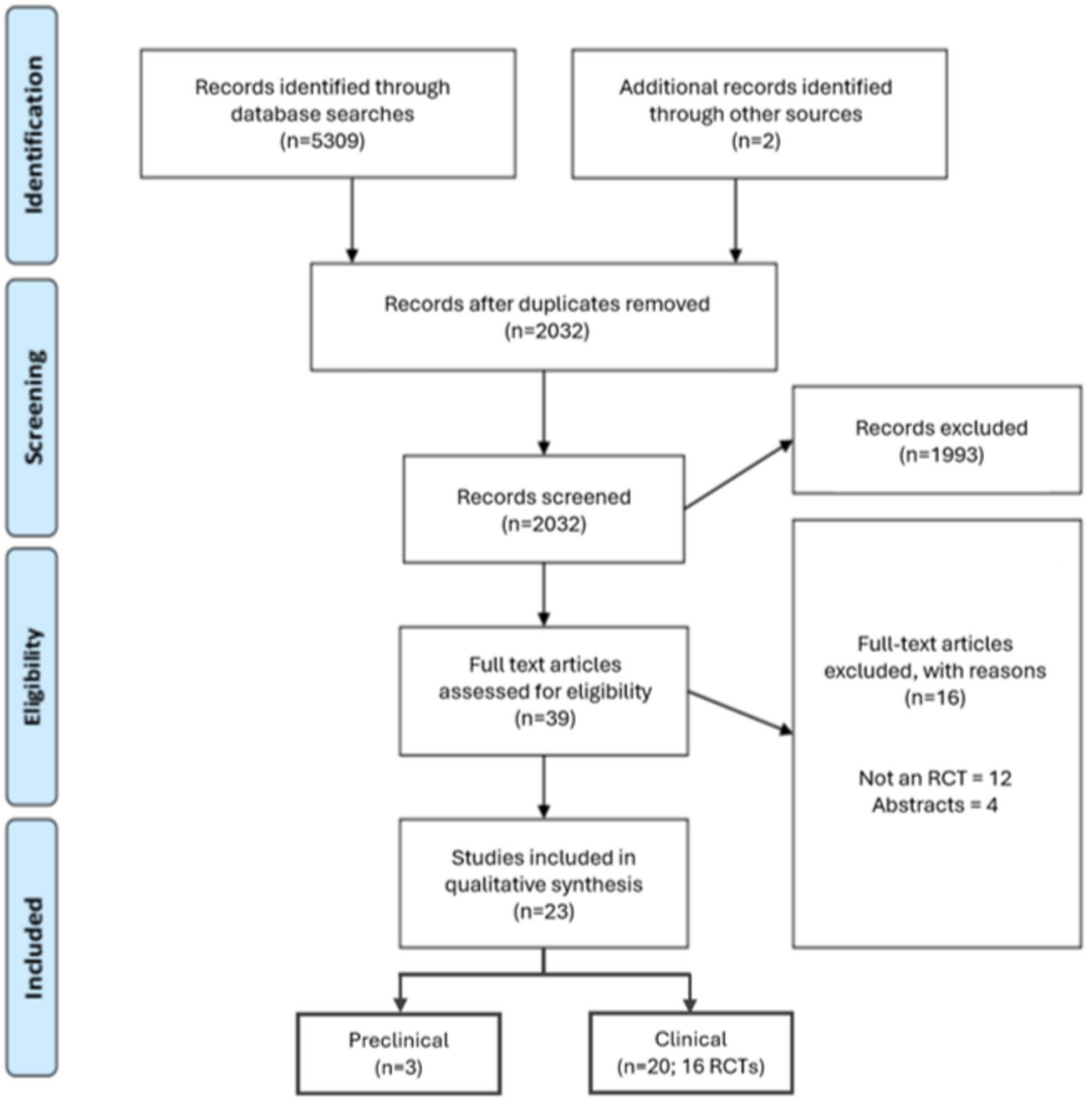
Study flow diagram

Initial searches identified 2,169 studies, of which 2032 remained after duplicates were removed. After screening title and abstracts 39 full text articles were identified for full review, of which 23 articles were finally included in the analysis. This composed of 3 preclinical (3 articles) and 16 clinical RCTs (20 articles, 4 of which reflected the same hip fracture clinical RCT with differing outcomes) as shown in the study flow diagram (Fig. 2).

### Quality assessment

Assessment of study methodological quality revealed very poor reporting of methods for randomisation, concealment of allocation, blinding and randomly selecting animals in preclinical studies (Table 1). In clinical studies, reporting of methods of allocation concealment was low (65% of studies) as was methods of blinding, particularly to those completing surgery (30%) and undertaking outcome assessments (65%) (Table 2).

**Table 1:** SYRCLEs risk of bias assessment for preclinical RCTs

Authors	Random allocation	Baseline characteristics	Allocation concealment	Animal housing	Caregiver blinding	Random animal selection	Blinded assessments	Completeness of data	Selective reporting	Other biases	Total Score
Catma et al. (2015) [28]	No	No	No	Yes	No	No	Yes	Yes	Yes	No	4
Zhou et al. (2017) [29]	No	No	No	Yes	No	No	No	Yes	Yes	No	3
Qiao et al. (2019) [30]	Yes	No	No	Yes	No	Yes	No	yes	Yes	No	5

**Table 2:** PEDro score for methodological quality of included clinical RCTs

Authors	Eligibility criteria	Random allocation	Allocation concealment	Baseline characteristics	Subject blinding	Interventionist blinding	Blinded assessments	Completeness of data	Intention to treat	Statistical comparisons	Measures of variability	Total Score
Memtsoudis et al. (2010) [31]	No	Yes	No	Yes	No	No	No	Yes	Yes	Yes	Yes	5
Oh et al. (2017) [32]	Yes	Yes	Yes	Yes	Yes	Yes	Yes	Yes	Yes	Yes	Yes	10
Murphy et al. (2010) [33]	Yes	Yes	Yes	No	Yes	No	No	No	No	Yes	Yes	5
Sha et al. (2014) [34]	Yes	Yes	Yes	No	Yes	No	No	No	No	Yes	Yes	5
Memtsoudis et al. (2014) [35]	Yes	Yes	No	Yes	Yes	No	Yes	Yes	Yes	Yes	Yes	8
Leurcharusmee et al. 2022a [36]	Yes	Yes	Yes	Yes	Yes	Yes	Yes	Yes	Yes	Yes	Yes	10
Leurcharusmee et al. 2022b [37]	Yes	Yes	Yes	Yes	Yes	Yes	Yes	Yes	Yes	Yes	Yes	10
Arikan et al. (2023) [38]	Yes	Yes	No	Yes	No	No	Yes	Yes	Yes	Yes	Yes	7
Sullivan et al. (2009) [39]	Yes	Yes	No	Yes	No	No	No	No	No	Yes	Yes	4
Koca et al. (2011) [40]	Yes	Yes	No	Yes	Yes	No	No	Yes	Yes	Yes	Yes	9
Orban et al. (2006) [41]	Yes	Yes	No	Yes	Yes	No	No	No	Yes	Yes	Yes	6
Lin et al. (2010) [42]	Yes	Yes	Yes	Yes	No	No	Yes	Yes	Yes	Yes	Yes	8
Van et al. (2008) [43]	Yes	Yes	No	Yes	Yes	No	No	Yes	Yes	Yes	Yes	7
Hu et al. (2010) [44]	Yes	Yes	Yes	Yes	Yes	Yes	Yes	Yes	Yes	Yes	Yes	10
Oh et al. (2020) [45]	Yes	Yes	Yes	Yes	Yes	Yes	Yes	Yes	Yes	Yes	Yes	10
Park et al. (2018) [46]	Yes	Yes	Yes	No	Yes	Yes	Yes	Yes	Yes	Yes	Yes	9
Ekeloef et al. (2019) [47]	Yes	Yes	Yes	Yes	No	No	Yes	Yes	Yes	Yes	Yes	8
Ekeloef et al. (2021a) [48]	Yes	Yes	Yes	Yes	No	No	Yes	Yes	Yes	Yes	Yes	8
Ekeloef et al. (2021b) [49]	Yes	Yes	Yes	Yes	No	No	Yes	Yes	Yes	Yes	Yes	8
Wahlstrøm et al. (2021) [50]	Yes	Yes	Yes	Yes	No	No	Yes	Yes	Yes	Yes	Yes	8
Total %	95%	100%	65%	85%	60%	30%	65%	80%	85%	100%	100%	

### Pre-clinical evidence

Three placebo-controlled studies evaluated RIC in rat models of fracture [29, 30, 30]. In total, 198 rats were used in protocols including RIPreC (n = 1) [28] and RIPostC (n = 2) [29, 30] interventions. One study also comparing RIPostC to intermittent hypoxia training (IHT) and a control group [30]. RIPreC was performed with seven cycles of five minutes intermittent pneumatic torniquet to the hind limb prior to fracture (timing pre-fracture not specified) [28]. RIPostC protocols included daily hind limb occlusion by torniquet for three cycles of 10 min for 7 or 28 days [29, 30]. Intervention characteristics and outcome measures are summarised in Table 3.

**Table 3:** Preclinical studies of RIC in bone healing following tibial fracture

Study	Population	%Male	Intervention	Numbers (control, intervention)	Type of RIC	Location	Measurements	Statistically significant results associated with RIC
Catma et al. (2015) [28]	Wistar-Hannover Rats	50	7 cycles of 5 min	48 (24, 24)	Pre (Timing not specified)	Hind limb (Ipsilateral)	Radiographs, histological evaluation, serum MDA levels	Increased callus maturity on histological analysis Lower MDA levels at the first week but not at the third and fifth week
Zhou et al. (2017) [29]	Sprague–Dawley Rats	100	3 cycles of 10 min daily for 7 days	64 (32, 32)	Post	Hind limb (Contralateral)	Radiographs, fracture zone for RT-qPCR, western blotting, immunohistochemistry, micro-CT and biomechanical testing	Increase in callus volume at day 14 and 28 on micro-CT Increased protein and mRNA expression of HIF-1α Up-regulation of downstream genes VEGF, Runx2, ALP and OCN protein Stronger fracture healing on biomechanical assessment
Qiao et al. (2019) [30]	Sprague–Dawley Rats	100	3 cycles of 10 min daily for 28 days Intermittent hypoxia training 5 min of 5 cycles post surgery	96 (32, 32, 32 IHT)	Post	Hind limb (Contralateral)	Radiographs, RT-qPCR analysis, western blotting, micro-CT and biomechanical testing	Increased markers of bone healing in IHT and RIPostC on micro-CT and radiographs Up-regulation of osteoblast expression markers VEGF, Runx2, ALP and OCN, as well as target gene HIF-1α, in the IHT and RIPostC

*IHT* = *intermittent hypoxia training; MDA* = *malondialdehyde; RT-qPCR* = *reverse transcription-quantitative polymerase chain reaction; micro-CT* = *micro-computed tomography; VEGF* = *vascular endothelial growth factor; Runx2* = *Runt-related transcription factor 2; ALP* = *alkaline phosphatase; OCN* = *osteocalcin; HIF-1α* = *Hypoxia-inducible factor-1alpha*

Outcome measures of bone healing such as callus volume and maturity were shown to be increased with RIC compared to controls in all three studies. Biomechanical assessment in both RIPostC studies also showed that RIC groups had stronger fracture healing than controls, although it was also greater in the IHT group [29, 30]. Serum malondialdehyde (MDA) levels, a marker of oxidative stress, were statistically lowered following RIPreC [28]. Osteoblast expression markers including vascular endothelial growth factor (VEGF), Runt-related transcription factor 2 (Runx2), alkaline phosphatase (ALP) and osteocalcin were upregulated, as well as the target gene Hypoxia-inducible factor-1alpha (HIF-1α), in the RIPostC studies compared to control (with the IHT also showing higher expression) [29, 30]. These findings suggest that the potential mechanisms of action on improved fracture healing, may be mediated via a reduction in oxidative stress and an enhanced osteoblastic response.

### Clinical evidence in elective orthopaedic surgery

Remote ischaemic conditioning has been investigated in elective orthopaedic surgery and 16 randomised controlled trials (RCTs) were reviewed (see Table 4) [32–46, 46]. Two manuscripts used the same participants and so were considered a single study [33, 34].

**Table 4:** Summary characteristics of RCTs of RIC in elective orthopaedic procedures

Authors	Type of study	Population	Intervention	Numbers (control, intervention)	Pressure	Location	Primary outcome	All reported outcome measures	Statistically significant results compared to placebo
Memtsoudis et al. (2010) [31]	Randomised, controlled	Total knee arthroplasty	1 cycle of 5 min	34 (17, 17)	250 mmHg	Operated limb	IL-6	Serum IL-6, CRP, TNF-alpha, leucocyte count. Urine desmosine levels Pain score, length of stay (LOS)	Improved pain score, reduced LOS
Oh et al. (2017) [32]	Randomised, controlled, double-blinded	Total knee arthroplasty	3 cycles of 5 min	72 (36, 36)	Double systolic blood pressure	Opposite thigh to operated limb	Regional cerebral oxygenation saturation (rScO_2_)	rScO_2_, ratio of the arterial oxygen partial pressure to the fractional inspired oxygen (PF ratio) HR, MAP Hct, lactate, Transfusion requirements, bleeding levels Serum CPK, LDH, AST, creatinine, IL-6, TNF-alpha, IL-10, TGF-beta Postoperative cognitive dysfunction (POCD) using confusion assessment method (CAM)	Higher HR, improved rScO_2_, improved PF ratio, reduced LDH, reduced transfusion requirements and bleeding levels
Murphy et al. (2010) [33]	Randomised, controlled, single-blinded	Total knee arthroplasty	3 cycles of 5 min	20 (10, 10)	100 mmHg above systolic blood pressure	Operated limb	Genomic response in muscle biopsies taken from the operative leg using microarray	Muscle biopsy and serum for gene expression profiles (micro-array and real time PCR) Hb, CRP, ESR, WCC IL-8, TNF-alpha, INF-gamma, IL-1-beta, IL-2, IL-10, IL-12, GM-CSF	Increase in expression of oxidative stress defence genes, immediate early response genes and mitochondrial genes. Upregulation of pro-survival genes was also observed and correlated with a downregulation of pro-apoptotic gene expression. Reduction in IL-6
Sha et al. (2014) [34]								Microarray expression profile from muscle biopsy	Down regulation of genes involved in neurological regulation of neuron apoptosis
Memtsoudis et al. (2014) [35]	Randomised, controlled, double-blinded	Total knee arthroplasty	1 cycle of 5 min	60 (30, 30)	250 mmHg	Operated limb	Postoperative pain using visual analogue scale (VAS)	VAS score, analgesic consumption Intraarticular fluid for TNF-alpha, IL-6 Periarticular circumference Muscle tissue oxygenation (by infrared spectroscopy) Prothrombin fragments F1/F2, d-dimer, Thrombin-antithrombin complex (TAT)	Reduced pain score at rest and with exercise
Leurcharusmee et al. 2022a [36]	Randomised, controlled, triple- blinded	Total knee arthroplasty	3 cycles of 5 min, CoQ10 28 days perioperatively	44 (10 control, 12 CoQ10, 14 RIPreC, 8 CoQ10 & RIPreC)	100 mmHg above systolic blood pressure	Operated limb	Mitochondrial oxygen consumption rates (OCRs) of peripheral blood mononuclear cells (PBMC) as a marker of oxidative phosphorylation	Venous blood PBMCs, postoperative pain scores using numeric rating scale (NRS) and morphine consumption	Increase in basal and ATP-linked respiration at two hours after reperfusion. (Morphine consumption was lower in CoQ10 group.)
Leurcharusmee et al. 2022b [37]	Randomised, controlled, double-blinded	Total knee arthroplasty	3 cycles of 5 min	24 (10, 14)	100 mmHg above systolic blood pressure	Operated limb	NR	Western blot analysis of muscle protein. Muscle strength. Health-related quality of life using the Thai version of EQ-5D	Increased mitofusin-2 protein and Opa1 protein expression. Preserved postoperative quadriceps muscle strength
Arikan et al. (2023) [38]	Randomised, controlled, double-blinded	Total knee arthroplasty	3 cycles of 5 min	60 (30, 30)	50 mmHg above systolic blood pressure	Upper arm	Total thiol-disulfide levels	Serum thiol-disulfide levels for thiol-disulfide homeostasis. Postoperative pain using VAS, nausea and vomiting (4 point scale)	Lower pain score at 15 th hour postop
Sullivan et al. (2009) [39]	Randomised, controlled, partial investigator-blinded	Cruciate ligament surgery	3 cycles of 5 min	25 (13, 12)	100 mmHg above systolic blood pressure (but not less than 250 mmHg)	Operated limb	NR	IL-2, IL-4, IL-6, IFN γ. T cell surface expression of CD45, CD62L and CD95. T cell CD4/CD8 and Th1/Th2 shifts	Reduced activation and proinflammatory cytokine production by CD4 cells, prevented CD4/CD8 derangement and lymphocyte directed immune dysfunction. Reduced serum IL-2
Koca et al. (2011) [40]	Randomised, controlled	Arthroscopic knee surgery	3 cycles of 5 min, 10 mg/kg intravenous N-acetylcysteine (NAC)	45 (15, 15, 15 NAC)	NR	NR	Serum malondialdehyde (MDA)	MDA, superoxide dismutase (SOD), glutathione peroxidase (GSH-Px), total antioxidant capacity (TAC), and total oxidant status (TOS)	Reduced mean serum MDA, TOS, SOD and GSH-Px levels
Orban et al. (2006) [41]	Randomised, controlled, single-blinded	Knee ligamentoplasty	1 cycle of 5 min, 1200 mg oral acetylcysteine	31 (11, 10, 10 acetylcysteine)	350 mmHg	Operated limb	Venous blood creatinine phosphokinase (CPK)	Myoglobin, CPK, potassium, phosphorus, lactate. Muscular strength of quadriceps of operated limb (by ASIA motor score). Morphine consumption, VAS	Lower morphine consumption in acetylcysteine and RIPreC groups
Lin et al. (2010) [42]	Randomised, controlled, double-blinded	Unilateral lower limb surgery	3 cycles of 5 min	30 (15, 15)	480 mmHg	Operated limb	Arterial-alveolar oxygen tension (a/A) ratio	Arterial blood gas, a/A ratio, alveolar-arterial oxygen tension difference (A-aDO2), respiratory index Plasma MDA, serum IL-6, IL-8, IL-10	Reduced change in arterial pO2, a/A ratio, A-aDO2 and respiratory index Reduced MDA, IL-6, IL-8
Van et al. (2008) [43]	Randomised, controlled, single-blinded	Lower limb surgery	3 cycles of 5 min	20 (10, 10)	300 mmHg	Operated limb	NR	Venous blood pH, partial oxygen pressure (PO2), partial carbon dioxide pressure (PCO2), lactate, potassium, sodium, glucose Lipid peroxidation using venous blood thiobarbituric acid reactive substances (TBARS) level HR, SpO2, MAP and spontaneous breathing rate (SRR)	No statistically significant findings between control and intervention
Hu et al. (2010) [44]	Randomised, controlled, triple-blinded	Cervical spondylosis decompression	3 cycles of 5 min	40 (20, 20)	200 mmHg	Right upper arm	Serum S-100B protein, serum neuron-specific enolase (NSE)	S-100B, NSE, median nerve somatosensory evoked potentials (SEPs), neurologic function recovery	Reduced serum S-100B and NSE, increased neurologic recovery rate
Oh et al. (2020) [45]	Randomised, controlled, double-blinded	Shoulder surgery	3 cycles of 5 min	63 (34, 29)	Double systolic blood pressure	Opposite thigh to operated side	Regional cerebral oxygenation saturation (rScO_2_)	rScO_2_, ratio of the arterial oxygen partial pressure to the fractional inspired oxygen (PF ratio) HR,MAP, Hct, lactate Serum IL-6, TNF-alpha, IL-10, TGF-beta	Higher rScO_2_
Park et al. (2018) [46]	Randomised, controlled, double-blinded	Orthopaedic surgery with history of IHD	3 cycles of 5 min	60 (30, 30)	250 mmHg or 50 mmHg above systolic blood pressure	Upper arm or calf that was not associated with the surgical field	Serum cardiac troponin I (cTnI) on day 1	Serum cTnI, creatine kinase (CK), creatine kinase myocardial band (CK-MB) ST-II segment of ECG lead II during surgery, and incidence of perioperative myocardial ischaemic events Creatinine, incidence of acute kidney injury (AKI)	No statistically significant findings between control and intervention

*IL-6* = *interleukin 6; CRP* = *C reactive protein; TNF-alpha* = *tumour necrosis factor alpha; HR* = *heart rate per minute; MAP* = *mean arterial blood pressure; LDH* = *lactate dehydrogenase; AST* = *aspartate aminotransferase; IL-10* = *interleukine 10; PCR* = *polymerase chain reaction; Hb* = *haemoglobin; ESR* = *erythrocyte sedimentation rate; WCC* = *white cell count; IL-8* = *interleukine 8; IL-2* = *interleukine 2; IL-12* = *interleukine 12; GM-CSF* = *granulocyte–macrophage colony stimulating factor; CoQ10* = *coenzyme Q10; IFN- γ* = *interferon gamma; CPK* = *creatine phosphokinase; SpO2* = *peripheral oxygen saturations; Hct* = *Haematocrit; NR* = *not reported. Other abbreviations already expanded in table*

In total, there were 628 participants, study sizes ranged from 20 to 72 participants. Thirteen studies included blinding methods, seven of these were double or triple blinded. Overall 12 of the 15 RCTs were rated good or excellent on PEDro quality rating [32, 36–38, 38, 41–50, 50]. The studies recruited participants undergoing total knee arthroplasty (n = 7) [32–38, 38], other populations of lower limb surgery (n = 5) [41–43, 43], cervical spondylosis decompression (n = 1) [44], shoulder surgery (n = 1) [45] and patients with a history of ischaemic heart disease undergoing orthopaedic surgery (n = 1) [46]. RIPreC in this context was considered safe with no studies reporting severe adverse events related to the intervention.

All RCTs used remote ischaemic preconditioning (RIPreC), described as being immediately prior to surgery, either before or after anaesthetic induction. RIPreC protocols included one (n = 3) [31, 35, 41] or three (n = 12) [33, 34, 34, 37–40, 40, 43–46, 46] cycles of five minutes of ischaemic conditioning. Pressures protocols varied and included double systolic blood pressure (SBP) (n = 2) [32, 45], 100 mmHg above SBP (n = 4) [33, 36, 37, 39], 50 mmHg above SBP (n = 1) [38] or ranged from 200 to 480 mmHg numerically (n = 7) [31, 32, 34, 35, 41–46, 46]. In lower limb surgery, nine out of the eleven studies which recorded the limb RIC was applied to, applied the cuff to the operated limb prior to torniquet for surgery. RIPreC is herein referred to simply as RIC. A primary outcome was documented in 12 studies [32–36, 36, 38, 41, 42, 42, 45, 46, 46] and can be seen in Table 4.

Markers of oxidative stress were measured in six trials [33, 34, 37,, 41, 43, 44] and were shown to be statistically significantly reduced in five [32, 33, 36, 40, 42] of these when comparing RIC to control. One study used micro-array from muscle biopsy of the operative leg to demonstrate a differential expression of 257 genes at the start of surgery and 786 genes one hour in to surgery. Some of the genes which were up-regulated were COX18, COX11, UCP3, TIMM10, MRPL43 and PDK4. Gene ontology analysis showed an increase in the expression of important oxidative stress defence genes, immediate early response genes and mitochondrial genes. There was also upregulation of pro-survival genes and a downregulation of pro-apoptotic genes in RIC treated participants [33].

Immune markers were measured by seven studies [32, 33, 33, 35, 39, 42, 45], however outcomes were varied with only three showing statistically significant changes [33, 39, 42]. Two of 6 studies measuring the inflammatory cytokine interleukine-6 (IL-6) demonstrated significant reductions with RIC [33, 42], while one of 2 studies measuring interleukin-8 (IL-8) demonstrated reductions [42]. Another study demonstrated a reduction in activation of CD4 cells, proinflammatory cytokine production IL-2, prevention of CD4/CD8 derangement and lymphocyte directed immune dysfunction [39].

Markers of neurone damage, dysfunction or recovery were measured by two studies [34, 44], both of which demonstrated statistically significant results. One study used micro-array to demonstrate downregulation of genes involved in neuronal apoptosis [34], the other showed increased neurologic recovery rate and a reduction in serum S-100B and neuron-specific enolase, early markers of neurologic dysfunction [44].

Two trials considered cerebral oxygenation [32, 45] and two measured peripheral oxygen levels [42, 43]. Regional cerebral oxygenation and venous and arterial measurements of partial pressure of oxygen (pO2) were statistically significantly increased in all four of the RIC groups compared to control.

Five studies reported on post-operative pain scores and analgesia consumption [31, 35, 36, 38, 41], four of which reported significantly lower levels amongst RIC treated groups [31, 35, 38, 41]. In one of these studies, the length of stay was also shorter in the intervention group compared with control [31]. Muscle strength was measured in two studies [37, 41], one of which demonstrated preservation of quadriceps strength in the RIC group.

### Clinical evidence in emergency orthopaedic surgery

Four manuscripts investigating RIC in individuals undergoing emergency orthopaedic surgery were identified. They all investigated hip fracture surgery in patients with cardiovascular risk factors, and their data were drawn from one RCT and its sub-studies. [48–50, 50]

The primary study recruited 648 patients with risk factors for cardiovascular disease across three centres. It was triple-blinded and included participants aged > 45 years with one of the four cardiovascular risk factors in Table 5.

**Table 5:** Characteristics of inclusion criteria in studies of RIC in people with cardiovascular risk factors undergoing emergency orthopaedic surgery

Inclusion criteria	Definition
Ischaemic heart disease	Angina pectoris, prior myocardial infarction, prior percutaneous coronary intervention or prior coronary artery bypass grafting
Peripheral arterial disease	Intermittent claudication, reduced peripheral arterial blood flow or previous vascular surgery due to peripheral arterial disease;
Previous Stroke	Prior history of ischaemic or haemorrhagic stroke
Cardiovascular risk factors	Age ≥ 70 years, congestive heart failure, previous transient ischaemic attack, diabetes and currently taking an oral hypoglycaemic agent or insulin, hypertension, preoperative serum creatinine concentration > 175 μmol/L, smoking within two years of surgery

RIC was delivered after induction of anaesthesia prior to surgery by electric tourniquet device to the upper arm. Four cycles of five minutes occlusion at 200 mmHg were used. The treatment was found to be safe with no adverse events related to RIC reported.

The primary outcome in this study was myocardial injury (defined by troponin rise within the first 4 days of surgery), with a secondary end point of major adverse cardiovascular events (MACE) [47]. Of the 648 that were randomised, 573 were included in the intention-to-treat analysis (286 RIC: 287 control). Results showed that there were statistically significant reductions in myocardial injury among RIC treated individuals (*p* = 0.002), as well as reductions in non-ischaemic causes for troponin rise, suggesting non-cardiac benefits to RIC in hip fracture patients. In the secondary outcomes, only the incidence of perioperative myocardial infarction within 30 days was reduced (*p* = 0.04). At one year follow-up, there was no difference between intervention and control groups in rates of death or readmission and no extra protective effect on vascular events was observed in the RIC treated group from 30 days onwards [48].

In a single-centre sub-study of this RCT [49] 38 participants (18 RIC: 20 control) had reactive hyperaemia indices measured by digital pulse amplitude tonometry to assess endothelial function at day 1 post-operatively. Endothelial dysfunction criteria were met in 18% of the RIC group and 40% of the control group indicating a beneficial effect from RIC, although this did not meet statistical significance. A further sub-study [50] evaluating the effects of RIC on thrombin generation, fibrinogen/fibrin turnover, plasminogen activation and fibrin structure pre-operatively and 2 h postoperatively, but did not find any differences between RIC and control groups.

## Discussion

The use of RIC in pre-clinical studies appears to improve bone healing and reduce oxidative stress. In clinical studies overall, the impact of RIC on orthopaedic outcomes appears promising, in particular with reference to reducing pain scores, analgesic use and reducing cardiovascular risk. RIC is safe, with no adverse outcomes recorded related to RIC in the studies reviewed. In elective orthopaedic surgery, a variety of populations were studied including bone and ligament surgery. Outcome measures were varied but included measures related to hypothesised mechanisms of RIC effect.

Data from RCTs of pharmacological interventions, such as calcitonin, bisphosphonates, and monoclonal antibodies for fracture healing are mixed [51]; such therapies have not yet made it into routine clinical practice. Physical therapies such as low-intensity pulsed ultrasound can produce osteoinductive effects and accelerate fracture healing and tensile strength [52], however accessibility of the therapy limits applicability and data for benefit in deep fractures is limited [53]. RIC is simple low cost and easy to implement. The preclinical evidence in this review highlighted the beneficial effect of RIC on bone healing, potentially mediated by a reduction in oxidative stress and enhanced osteoblastic activity [29, 30, 30]. Formation of reactive oxygen species (ROS) in tissues in response to stress can impair the function of vital metalloenzymes in cells leading to inflammation, as well as the integrity of DNA and RNA itself, ultimately affecting cell function [54]. Organisms have thus evolved scavenging and repair systems in order to keep ROS in check. The mitigating effect that RIC has on ROS and oxidative stress has also been demonstrated in animal models of ischaemic stroke, possibly related to upregulation of nuclear factor-E2-related factor 2/heme oxygenase-1 pathway (Nrf2/HO-1) that plays a crucial role in upregulating expression of various antioxidant defence and anti-inflammatory genes [55]. The study by Sha et al. included in this review similarly revealed that a single dose of RIC led to upregulation of genes involved in ROS defence mechanisms in the muscle [34]. Identification of potentially relevant genes profiles and increasing sophistication and accessibility of genetic analysis techniques will hopefully mean that gene profiling as an outcome marker in RIC studies becomes increasingly common and reveals a clearer understanding of which pathways are implicated in RIC protection. Further, effects on oxidative stress may be mediated by alterations to mitochondrial energy metabolism in response to RIC. Lv et al. (2020) showed in preclinical models of cerebral ischaemia that RIC preserved mitochondrial respiratory chain function in the brain and ameliorated apoptosis via endogenous mitochondrial pathways [56]. Not only does this represent another mechanism that alters oxidative stress response, but it may explain the attenuated adenosine triphosphate (ATP) depletion that occurs in RIC treated porcine skeletal muscle following experimental ischaemic stress [20].

Data on the effects of RIC on inflammatory mediator profiles were inconsistent from the studies included in this review. IL-6 was the most commonly evaluated in orthopaedic studies of RIC. It is a pleotropic cytokine secreted by T cells and macrophages to activate the immune response during infection or trauma [57] and is a marker of the proinflammatory response. Animal studies investigating the effect of RIC in experimental myocardial infarction [58, 59] have demonstrated that RIC, whether completed pre, per or post ischaemia, can lead to reductions in circulating IL-6. However, many clinical studies of ischaemic heart disease have not reproduced similar reductions [61, 62, 62]. Indeed, some clinical studies (renal transplant recipients) have reported increased levels of IL-6 in response to RIC [63], and so our understanding of the role IL-6 plays in inflammation may be oversimplified. The fact that IL-6 also acts as an osteoclast differentiation modulator, often involved in bone remodelling [64] may explain variations we observed in response to RIC in the in this review, as bone remodelling pathways are often activated in orthopaedic procedures. Further, variation in the type of surgery conducted, patient comorbid diseases and method of anaesthesia may also affect and confound inflammatory response and may have added to the variation in response seen in these studies.

Many orthopaedic procedures are conducted under general anaesthetic, associated with risk of impairments in pulmonary oxygenation, and cardiovascular and cerebrovascular events. RIC may mitigate such complications through its effects on vascular endothelial function and tissue perfusion. The endothelium of blood vessels plays a crucial role in vascular homeostasis by regulating vascular tone, releasing vasodilators and mediating platelet aggregation [65]. Impairments in endothelial function commonly occur following episodes of ischaemia and reperfusion, such as those occurring following myocardial infarction, stroke or even prolonged application of tourniquets [66]. RIC is thought to protect against such endothelial injury in humans via glucagon-like peptide-1 receptor-mediated pathways [67]. Such preservation of endothelial function may is implicated in improved cerebral perfusion and oxygenation [68] as well as pulmonary gas exchange in ventilated patients [69] following RIC. Markers of cerebral oxygenation and peripheral partial pressures of oxygen were improved in RIC RCTs of total knee arthroplasty, lower limb surgery and shoulder surgery in this review. Cardioprotection following hip fracture surgery seen within the first 30 days [46] may be presumed to be related to endothelial preservation in part and is suggested from FMD sub-studies [49], but may also be related to preservation of mitochondrial function as previously demonstrated in clinical studies of RIC in coronary artery bypass surgery [70].

Of interest was the finding that RIC helped reduce pain scores and analgesia use following orthopaedic surgery. The relationship between RIC and pain is somewhat obscure. However, the central nervous system modulates nociceptive input from peripheral tissues, and the autonomic nervous system play a crucial role in this modulation [71]. It is known that RIC can influence the autonomic nervous system [72] and as such may also modulate the way nociceptive inputs are perceived. Although it is also possible that reduced levels of inflammation and tissue injury as a result of RIC may reduce pain and analgesia requirements. Studies are currently underway to evaluate the effect of RIC on pain in women with osteoarthritis [23].

This review has highlighted that a single dose of RIC delivered before orthopaedic procedures can result in a variety of beneficial effects on inflammation, organ function and pain as well as mitigate against common cardiovascular complications. RIC protocols used varied significantly in duration, frequency, pressure and limb conditioned. Further work on identifying the optimal dosing strategy of RIC delivery is still required in this cohort of patients. Indeed, weather repeated doses of RIC post procedure (RIPostC) may add further benefit is yet to be established. RIC may have immediate effects such as promoting endothelial release of vasodilating substances, and late effects such as upregulation of transcription factors that lead to expression of various proteins and enzymes involved in oxidative pathways and mitochondrial function [73]. Ekelof et al. in the PIXIE trial of hip fracture only identified an early protective effect of RIC on MACE which could be a result of the fact that they applied only a single dose at anaesthesia induction [46]. It may be that further doses of RIC conditioning post-operatively may have led to further cardioprotective effects at long term follow up and requires further investigation.

This review has a number of limitations. Firstly, there were 20 clinical manuscripts identified deriving data from 16 RCTs, only one of which included patients undergoing emergency surgery. Thus it is difficult to generalise this data to emergency orthopaedic surgery cohorts. Second, there was significant heterogeneity in types of orthopaedic procedure undertaken (including duration of limb tourniquets applied) and the protocols of RIC used, which may account for some of the variation in outcomes seen. From the data included in this review, it is unclear if a greater pressure, ischaemic duration, or number of cycles results in a more profound physiological effect following RIC. These differing treatment parameters require further exploration. Furthermore, all included clinical studies investigated RIC delivered manually using a sphygmomanometer. Development of automated devices may offer ease of use for clinical staff, but would need to be balanced against cost. Additionally it is unclear whether surgical procedures operating under a tourniquet (further ischaemic stimulus) influences effects of RIC. However, 9 of the included clinical RCTs involved lower limb procedures operating under tourniquet conditions, many of whom reported physiological effects follow RIC compared to sham, suggesting additional benefit of ischaemia reperfusion cycles prior to surgery itself. Third, where general anaesthetics were used for procedures, types of anaesthetic agent were not always specified, and it is known that some anaesthetic agents can influence the effects of RIC. For example, preclinical models investigating the cardioprotective effects of RIC have shown that propofol negates the reduction in myocardial infarct size when compared to using sevoflurane or pentobarbital [74]. This may be due to the inhibitory effects of propofol on signal transducer pathways (e.g. signal transducer activator of transcription 5, stat5) or how it influences gaba-aminobutyric acid mediated vagal nerve activation [75]. Fourth, although participant characteristics were reported in many studies, very few included very elderly individuals (aged > 75 years for example) and those with multiple comorbidities, which makes generalisation to these cohorts also difficult, although our ageing population means these are the types of individuals we will see in clinical practice. Future studies should ensure reporting of comorbid diseases such as diabetes, especially as conditions such as these, and potentially their treatments (e.g. sulphonylureas) may attenuate the effects of RIC [76]. Fifth, no studies included in this review used biomarkers to guide RIC therapy or monitor for treatment responses. While biomarkers of RIC have been proposed [77], they require further study before they can be used reliably to identify responders from non-responders, or guide the intensity of how RIC is delivered. Finally, bar the PIXIE trial, most of the included studies were small (n < 60) and as such, are prone to small study bias. Future studies should aim to be powered for clinically meaningful outcomes such as return to function and pain as primary outcomes. Long-term follow up is needed to assess functional recovery, bone healing and quality of life, as well as incorporating mechanistic evaluation as secondary measures, in order to understand whether RIC will eventually move from research intervention to clinical practice.

## Conclusion and future directions

RIC is a safe, simple and economical therapy which has been shown to have promising effects in pre-clinical and clinical models of orthopaedic surgery. Pre-clinical work suggests enhancing effects on bone healing while clinical studies suggest positive effects on oxidative stress, inflammation, endothelial and vascular function, as well as clinical parameters such as cardiovascular complications, pain and analgesia use.

## Supplementary Information

Additional file 1. 

Additional file 2. 

## Data Availability

The datasets generated during the current systematic review are available from the corresponding author on reasonable request.

## Declarations

### Protocol registration

This systematic review protocol was not registered or previously published.
