# Supplementary material for: Role of remote ischaemic conditioning in fracture healing and orthopaedic surgery—a systematic review and narrative synthesis
Source: J Orthop Surg Res. 2025 May 7;20:448. doi: 10.1186/s13018-025-05772-6 (PMC12060424; doi:10.1186/s13018-025-05772-6)
Supplement: Supplementary file 2 — Additional file 2. [file 13018_2025_5772_MOESM2_ESM.docx]

**Additional file 1. Search strategy for use in OVID MEDLINE and EMBASE**

| **MEDLINE** |  |
| --- | --- |
| ischaemic conditioning OR remote ischaemic conditioning OR perconditioning OR preconditioning OR postconditioning | 28,334 |
| Trauma OR bone injury OR fracture OR hip fracture OR break OR fragility OR bone healing | 1,816,075 |
| Musculoskeletal OR orthopaedics OR trauma OR ligament OR meniscus OR elective OR emergency OR operative OR tissue OR muscle OR cartilage | 12,084,927 |
| ((Musculoskeletal OR orthopaedics OR trauma OR ligament OR meniscus OR elective OR emergency OR operative OR tissue OR muscle OR cartilage) AND (Trauma OR bone injury OR fracture OR hip fracture OR break OR fragility OR bone healing) AND (ischaemic conditioning OR remote ischaemic conditioning OR preconditioning OR perconditioning OR postconditioning) | 2,204 |

| **EMBASE** |  |
| --- | --- |
| ischaemic conditioning OR remote ischaemic conditioning OR perconditioning OR preconditioning OR postconditioning | 6,503 |
| Trauma OR bone injury OR fracture OR hip fracture OR break OR fragility OR bone healing | 1,765,344 |
| Musculoskeletal OR orthopaedics OR trauma OR ligament OR meniscus OR elective OR emergency OR operative OR tissue OR muscle OR cartilage | 12,213,785 |
| ((Musculoskeletal OR orthopaedics OR trauma OR ligament OR meniscus OR elective OR emergency OR operative OR tissue OR muscle OR cartilage) AND (Trauma OR bone injury OR fracture OR hip fracture OR break OR fragility OR bone healing) AND (ischaemic conditioning OR remote ischaemic conditioning OR preconditioning OR perconditioning OR postconditioning) | 3,105 |
